# Supplementary material for: Loss of polarity regulators initiates gasdermin-E-mediated pyroptosis in syncytiotrophoblasts
Source: Life Sci Alliance. 2023 Jul 19;6(10):e202301946. doi: 10.26508/lsa.202301946 (PMC10355286; doi:10.26508/lsa.202301946)
Supplement: Supplementary file 1 [file LSA-2023-01946_SdataF2_F4_F5_FS3_FS5_FS12.pptx]

## Slide 1
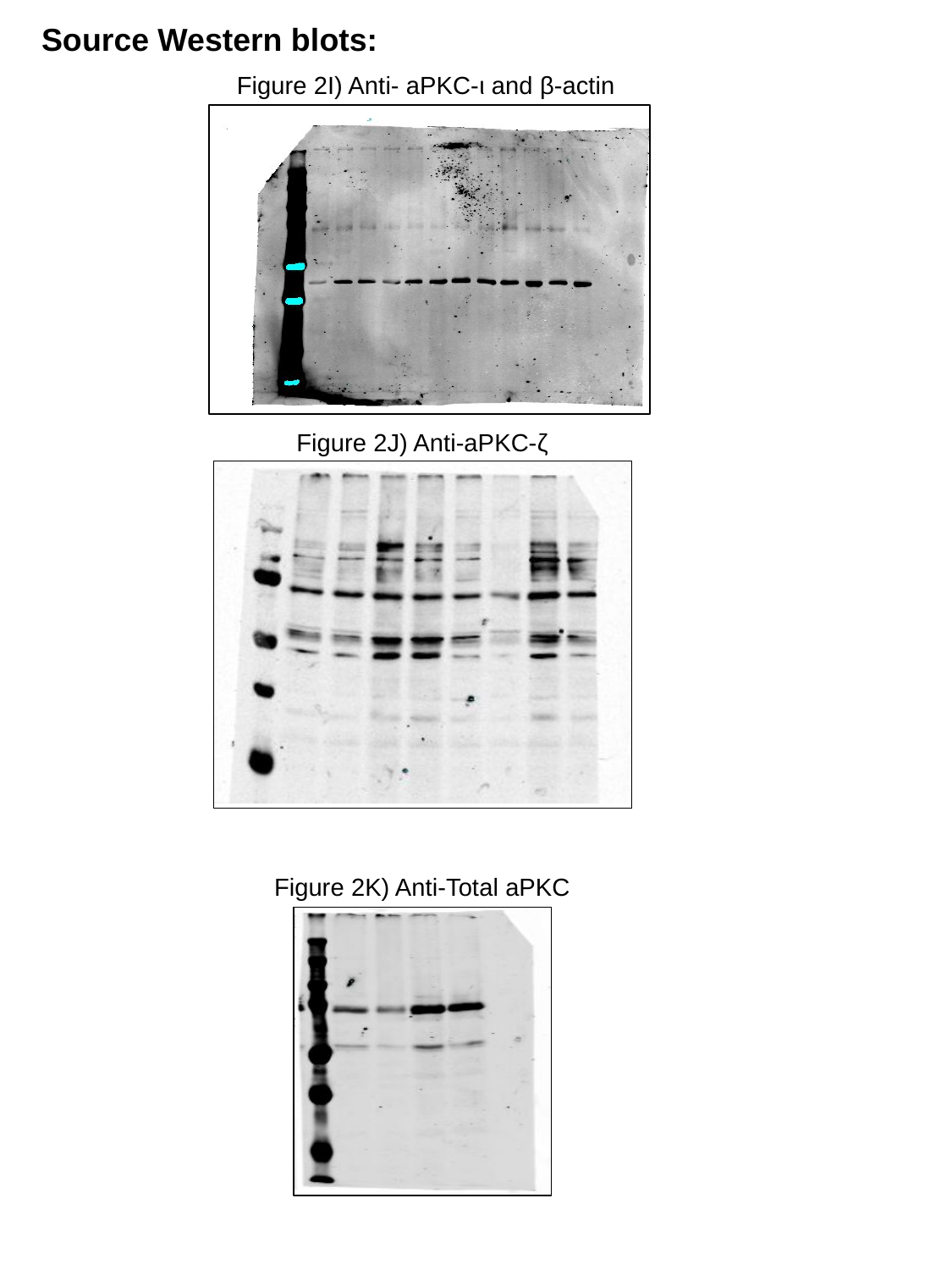

Source Western blots:
 Figure 2I) Anti- aPKC-ι and β-actin
Figure 2J) Anti-aPKC-ζ
Figure 2K) Anti-Total aPKC

## Slide 2
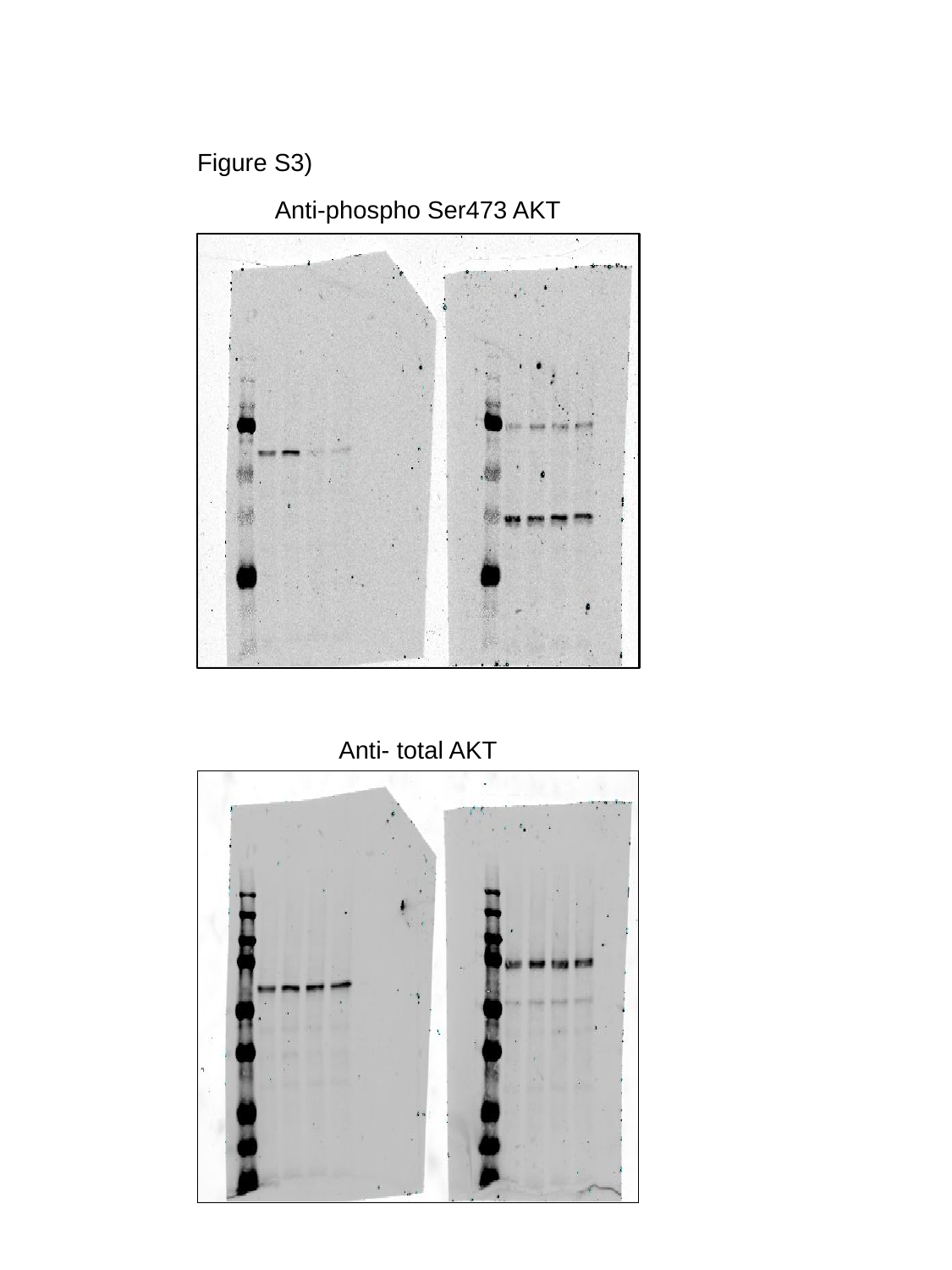

Figure S3)
Anti-phospho Ser473 AKT
Anti- total AKT

## Slide 3
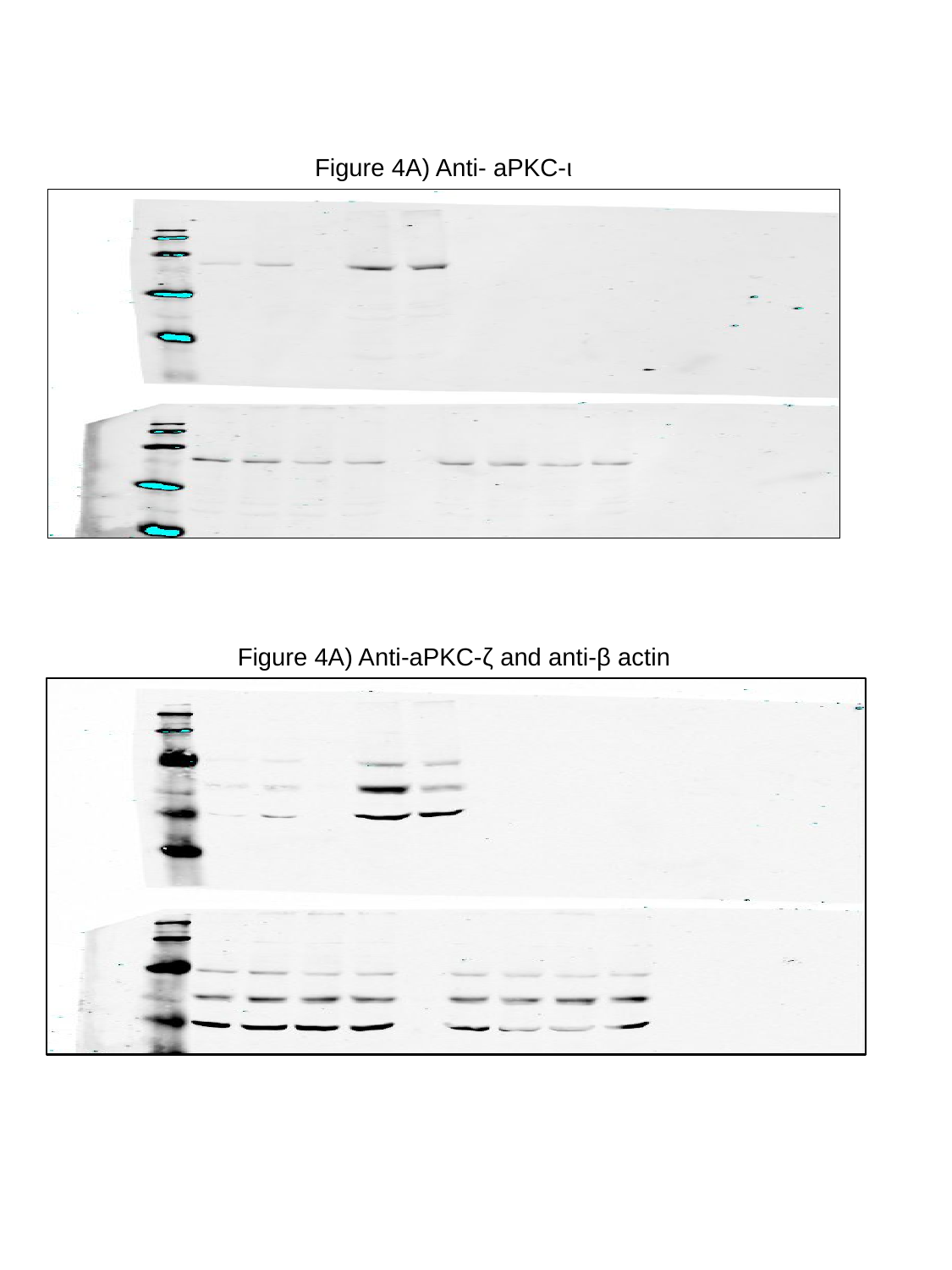

Figure 4A) Anti- aPKC-ι
Figure 4A) Anti-aPKC-ζ and anti-β actin

## Slide 4
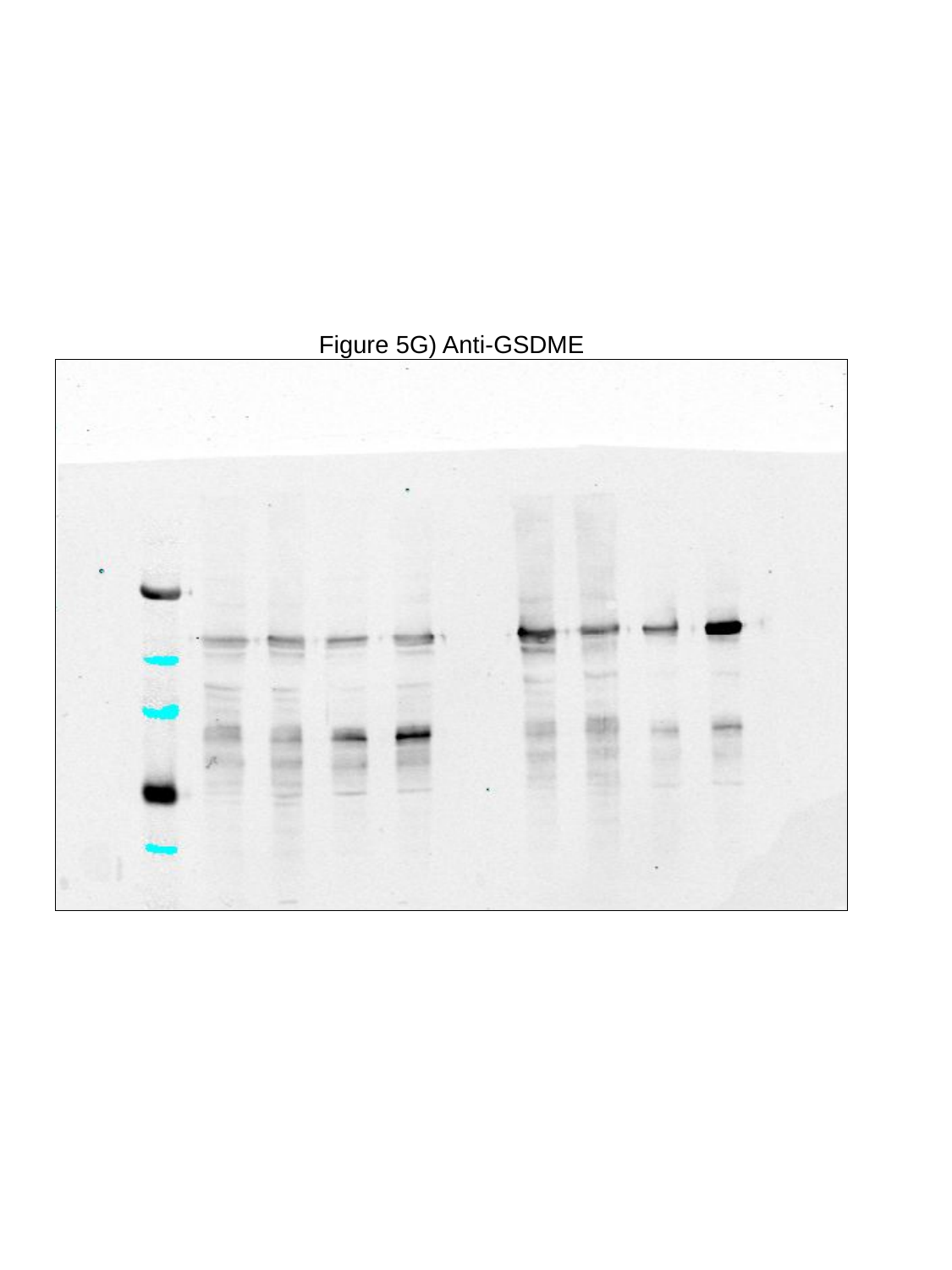

Figure 5G) Anti-GSDME

## Slide 5
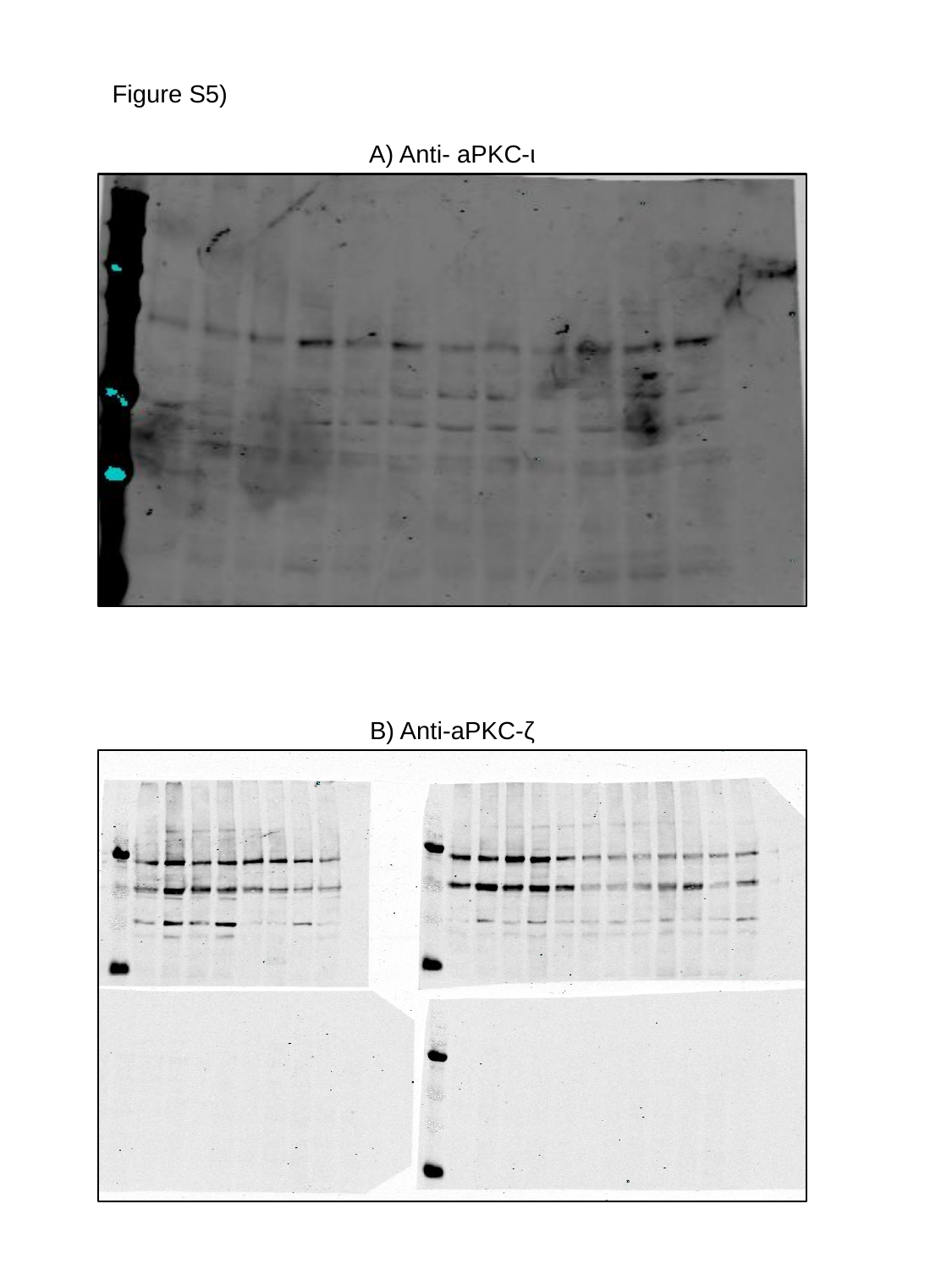

Figure S5)
A) Anti- aPKC-ι
B) Anti-aPKC-ζ

## Slide 6
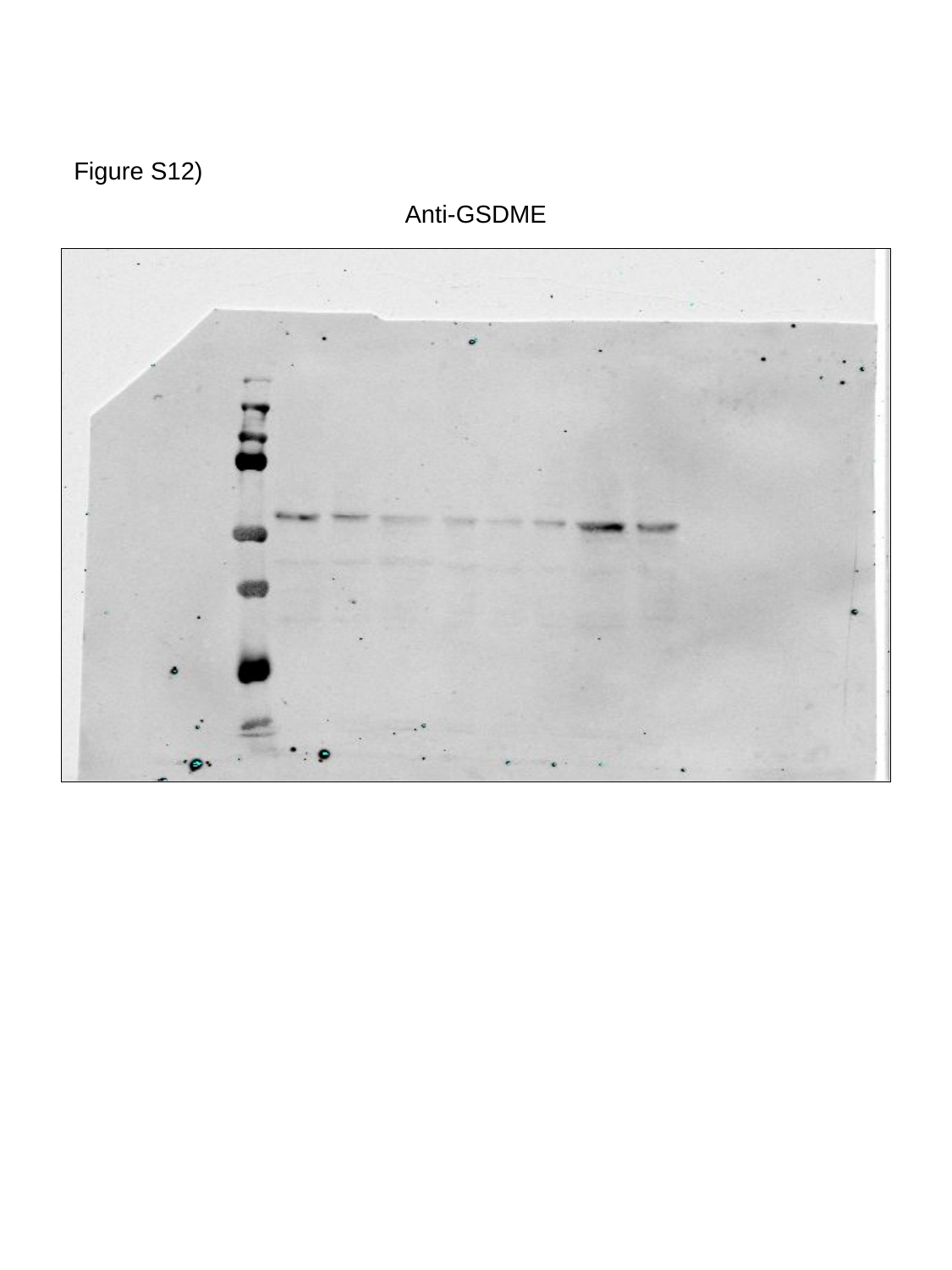

Figure S12)
Anti-GSDME
